# Biomarker analysis to predict the pathological response to neoadjuvant chemotherapy in locally advanced gastric cancer: An exploratory biomarker study of COMPASS, a randomized phase II trial

**DOI:** 10.18632/oncotarget.27658

**Published:** 2020-07-28

**Authors:** Takashi Oshima, Takaki Yoshikawa, Yohei Miyagi, Satoshi Morita, Michio Yamamoto, Kazuaki Tanabe, Kazuhiro Nishikawa, Yuichi Ito, Takanori Matsui, Yutaka Kimura, Tomoyuki Yokose, Yukihiko Hiroshima, Toru Aoyama, Tsutomu Hayashi, Takashi Ogata, Haruhiko Cho, Yasushi Rino, Munetaka Masuda, Akira Tsuburaya, Junichi Sakamoto

**Affiliations:** ^1^Department of Gastrointestinal Surgery, Kanagawa Cancer Center, Asahi-ku, Yokohama, Kanagawa 241-8515, Japan; ^2^Department of Gastric Surgery, National Cancer Hospital, Chuo-ku, Tokyo 104-0045, Japan; ^3^Kanagawa Cancer Center Research Institute, Asahi-ku, Yokohama, Kanagawa 241-8515, Japan; ^4^Department of Biomedical Statistics and Bioinformatics, Kyoto University Graduate School of Medicine, Sakyo-ku, Kyoto, Kyoto 606-8507, Japan; ^5^Graduate School of Environmental and Life Science, Okayama University, Kita-ku, Okayama, Okayama 700-8530, Japan; ^6^Department of Gastroenterological and Transplant Surgery, Hiroshima University, Minami-ku, Hiroshima, Hiroshima 734-8551, Japan; ^7^Department of Surgery, National Hospital Organization Osaka National Hospital, Chuo-ku, Osaka, Osaka, 540-0006, Japan; ^8^Department of Gastroenterological Surgery, Aichi Cancer Center Hospital, Chikusa-ku, Nagoya, Aichi 464-8681, Japan; ^9^Department of Surgery, Aichi Cancer Center, Aichi Hospital, Kakemachi, Okazaki, Aichi 444-0011, Japan; ^10^Department of Surgery, NTT West Japan Osaka Hospital, Tennouji-ku, Osaka, Osaka 543-0042, Japan; ^11^Department of Surgery, Yokohama City University, Kanazawa-ku, Yokohama, Kanagawa 236-0004, Japan; ^12^Department of Gastric Surgery, Tokyo Metropolitan Cancer and Infectious Diseases Center Komagome Hospital, Bunkyo-ku, Tokyo 113-8677, Japan; ^13^Department of Pathology, Kanagawa Cancer Center, Asahi-ku, Yokohama, Kanagawa 241-8515, Japan; ^14^Department of Surgery, Ozawa Hospital, Odawara, Kanagawa 250-0012, Japan; ^15^Tokai Central Hospital, Kakamigahara, Gifu 504-8601, Japan

**Keywords:** gastric cancer, neoadjuvant chemotherapy, pathological response, predictive biomarkers, personalized therapy

## Abstract

Background: The findings of COMPASS, a randomized phase II study, suggested that the regimens and courses of neoadjuvant chemotherapy (NAC) for locally advanced gastric cancer (GC) did not affect the pathological response. However, pathological complete response was achieved in 10% patients who received four courses of either S-1/cisplatin or paclitaxel/cisplatin. We hypothesized that if relevant biomarkers could be used to predict the suitable NAC regimen before treatment initiation, further improvements could be ensured in the outcomes of locally advanced GC.

Materials and Methods: mRNA extraction, real-time polymerase chain reaction, and immunohistochemical analyses were performed using endoscopic biopsy specimens of primary tumors, collected prior to NAC, to determine the clinically relevant biomarkers.

Results: *TIMP1*, *DSG2*, *RRM1*, *MUC2*, *EGFR*, *ZDHHC14*, and *CLDN18.2* were identified as biomarker candidates, since their expression was significantly associated with the pathological responses to each NAC regimen. Furthermore, *TIMP1* and *DSG2* were identified as predictive biomarkers of the pathological response to each NAC regimen.

Conclusions: The effective prediction of the pathological response to NAC regimens in locally advanced GC using biomarkers identified from endoscopic biopsy specimens indicates the possibility of personalizing NAC based on biomarker analysis.

## INTRODUCTION

Gastric cancer (GC) is a disease of global relevance. With an estimated 1 million new cases each year, it is the fifth most commonly diagnosed malignancy worldwide. In 2018, 784,000 people died worldwide, making it the third leading cause of cancer-related deaths [1]. Currently, standard treatments for locally advanced GC in Asia, Europe, and the United States include curative gastrectomy followed by adjuvant chemotherapy, surgery with pre- and postoperative chemotherapy, and surgery with postoperative chemoradiotherapy [2–6]. Recent phase III trials [2–4] revealed that adjuvant chemotherapy after curative gastrectomy improved the overall survival (OS) in patients with locally advanced GC. However, even after curative resection, the outcomes of adjuvant chemotherapy administered after gastrectomy are insubstantial [7, 8], and chemotherapy of greater intensity is necessary to further improve the survival rates.

Neoadjuvant chemotherapy (NAC) is considered effective for the treatment of stage III locally advanced GC. NAC is a form of multidisciplinary treatment in which chemotherapy is used initially to reduce the tumor size and eliminate micro-metastases, following which the remaining primary and metastatic lesions are excised. The advantages of neoadjuvant chemotherapy include a high rate of R0 resection, tumor regression, high compliance, and the avoidance of unnecessary surgery [9]. However, positive results are yet to be achieved in phase III studies on NAC conducted thus far. Although the results of two-phase III randomized studies on NAC in locally advanced GC, JCOG0501 [10] and PRODIGY [11], were recently reported, similar to the results from previous trials, there were no significant differences in OS between patients who received and did not receive NAC.

A randomized phase II study (COMPASS) was conducted to determine the optimal regimen and the number of courses of NAC, and to compare the effectiveness of two or four courses of NAC with that of S-1/cisplatin (SC) or paclitaxel/cisplatin (PC) in 83 patients with locally advanced GC using a two-by-two factorial design. The results revealed that there was no significant difference between the pathological responses to SC and PC or those to two and four courses of NAC. However, it was noteworthy that pathological complete response was achieved in 10% of the patients who received four courses of either SC or PC [12]. This suggested that a significant pathological response might be achieved by administering an appropriate regimen of NAC for a certain time period; that is, if the pathological response to each NAC regimen can be predicted prior to the initiation of therapy and the personalization of NAC can be facilitated, the outcomes could be expected to improve. This study on COMPASS trial biomarkers investigated the relevant biomarkers that could predict the pathological response to each NAC regimen in locally advanced GC.

## RESULTS

### Biomarker study cohort

**Figure 1:**
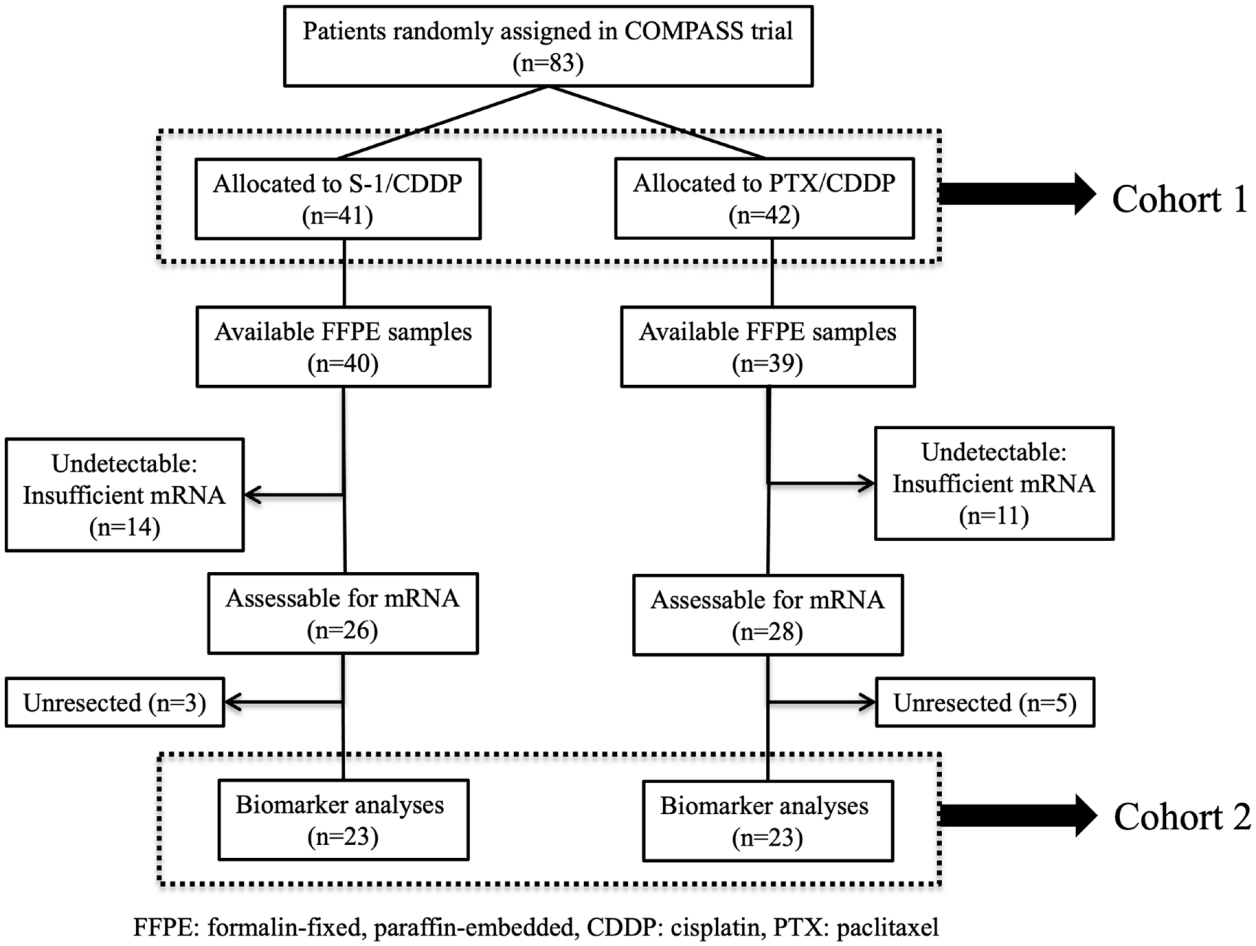
CONSORT diagram. Among the 83 patients who participated in the COMPASS trial, mRNA extraction was performed successfully for 78 patients (94%) and the expression levels of 127 genes were measurable in 46 patients (55%). Twenty-three patients received SC; the remaining 23 received PC.

A CONSORT diagram is presented in Figure 1. Among the 83 patients enrolled in the COMPASS trial, 41 and 42 patients were assigned to receive SC and PC, respectively (cohort 1). Seventy-nine GC tissue specimens that had been obtained endoscopically from each patient before NAC initiation were collected, and 46 samples were further used in the biomarker study (cohort 2). Although stage IV patients with peritoneal dissemination or non-local lymph nodes metastasis did not undergo resection in this study, four patients with no peritoneal metastasis and peritoneal lavage cytology positive (P0 CY1) underwent gastrectomy with D2 lymph node resection. These procedures are adopted from previous reports of positive outcomes in patients who underwent gastrectomy with D2 lymph node dissection followed by chemotherapy with S-1 for P0 CY1 GC [13], as well as on Japanese GC treatment guidelines (ver. 5). The outcomes in the patients with P0 CY1 included in this study were considerably encouraging. Therefore, in this study, the pathological response in primary tumors was evaluated using specimens obtained from patients, including those with P0 CY1.

The patient characteristics and pathological responses in the primary tumors were then compared between cohort 1 and 2 in the SC and PC arms. There was no different between the patient characteristics of cohorts 1 and 2 in the SC or PC arms, (Table 1). Furthermore, there was no significant difference in the patient characteristics between the cohorts in the SC or the PC arms (Table 2).

**Table 1:** Patient characteristics

Variables/categories		S-1/cisplatin arm				Paclitaxel/cisplatin arm			
		Cohort 1 (*n* = 41)		Cohort 2 (*n* = 23)		Cohort 1 (*n* = 42)		Cohort 2 (*n* = 23)	
		No.	%	No.	%	No.	%	No.	%
Age (years)	Median (range)	65 (32–79)^*^		66 (41–79)^*^		66 (43–80)^*^		65 (44–76)^*^	
Gender	Male	26	63	15	65	32	76	19	83
	Female	15	37	8	35	10	24	4	17
Performance status	0	41	100	23	100	40	95	23	100
	1	0	0	0	0	2	5	0	0
Macroscopic type	Non-scirrhous	30	73	14	61	27	64	14	61
	Type 4/giant type 3	11	27	9	39	15	36	9	39
Histological type	Differentiated	17	41	7	30	19	45	11	48
	Undifferentiated	24	59	16	70	23	55	12	52
Clinical T	T2	0	0	0	0	1	2	0	0
	T3	2	5	1	4	4	10	1	4
	T4a	36	88	20	87	32	76	21	91
	T4b	3	7	2	9	5	12	1	4
Clinical N	N0	5	12	4	17	7	17	5	22
	N1	19	46	11	48	16	38	8	35
	N2	17	41	8	35	18	43	9	39
	N3	0	0	0	0	1	2	1	4
Clinical M	Negative	35	85	21	91	34	81	21	91
	Positive	6	15	2	9	8	19	2	9
Site of M	CY1^**^ and P0^***^	6	100	2	100	7	88	2	100
	Para-aortic nodes	0	0	0	0	1	12	0	0

^*^Expressed as median (range), ^**^CY1; positive for peritoneal lavage cytology, ^***^P0; negative for peritoneal dissemination.

**Table 2:** Pathological response in primary tumors based on criteria proposed by the MD Anderson Cancer Center

Pathological response (grade)	S-1/cisplatin arm				Paclitaxel/cisplatin arm			
	Cohort 1 (*n* = 41)		Cohort 2 (*n* = 23)		Cohort 1 (*n* = 42)		Cohort 2 (*n* = 23)	
	No.	%	No.	%	No.	%	No.	%
I	6	15	3	13	4	10	1	4
IIa	15	37	8	35	19	45	9	39
IIb	11	27	9	39	12	30	11	48
III	4	10	1	4	0	0	0	0
IV	2	5	2	9	2	5	2	9
Unknown	0	0	0	0	0	0	0	0
Unresected	3	7	–	–	5	12	–	–
IIb–IV	17/41	41%	12/23	52%	14/42	33%	13/23	57%

### Biomarker candidates at the mRNA level that predict the pathological response of locally advanced GC to each NAC regimen

The following genes were denoted as biomarker candidates: metallopeptidase inhibitor 1 (*TIMP1*), desmoglein-2 (*DSG2*), ribonucleotide reductase catalytic subunit M1 (*RRM1*), mucin-2 (*MUC2*), epidermal growth factor receptor (*EGFR*), DHHC-type palmitoyl transferase 14 (*ZDHHC14*), and claudin-18 isoform 2 (*CLDN18.2*); the expression profiles of these genes had a significant association with pathological responses to SC or PC.

When biomarker candidates at the mRNA level were divided into high and low expression biomarkers based on the cutoff values determined using statistical analysis methods, (as shown in materials and methods), the high expression of *TIMP1*, *DSG2*, *RRM1*, and *MUC2*, and the low expression of *EGFR*, *ZDHHC14*, and *CLDN18.2* appeared to be associated with a better pathological response to SC. In addition, the high expression of *EGFR*, *ZDHHC14*, and *CLDN18.2* and the low expression of *TIMP1*, *DSG2*, *RRM1*, and *MUC2* were associated with a better pathological response to PC (Table 3, Supplementary Table 1).

**Table 3:** Biomarker candidates for predicting the pathological response to NAC with SC or PC

Biomarker	Category	SC arm			PC arm			Comparison between the treatment groups *P*-value	*P*-value for treatment interaction
		No.	Responders		No.	Responders			
			No.	%		No.	%		
***ZDHHC14***	< 0.608	8	7	87.5	7	1	14.3	0.0101	**0.0002**
	≧0.608	15	5	33.3	16	12	75.0	0.0319	
***TIMP1***	< 10.473	18	7	38.9	20	13	65.0	0.1927	**0.0013**
	≧10.473	5	5	100	3	0	0.0	0.0179	
***CLDN18.2***	< 23.564	11	8	72.7	17	7	41.2	0.1367	**0.0016**
	≧23.564	12	4	33.3	6	6	100	0.0128	
***EGFR***	< 0.549	13	9	69.2	12	4	33.3	0.1152	**0.0028**
	≧0.549	10	3	30.0	11	9	81.8	0.0299	
***RRM1***	< 0.803	15	7	46.7	18	13	72.2	0.1686	**0.0075**
	≧0.803	8	5	62.5	5	0	0.0	0.0754	
***MUC2***	< 14.04	18	7	38.9	19	12	63.2	0.1939	**0.0077**
	≧14.04	5	5	100	4	1	25.0	0.0476	
***DSG2***	< 4.312	10	3	30.0	12	9	75.0	0.0836	**0.0091**
	≧4.312	13	9	69.2	11	4	36.7	0.2173	

Abbreviations; SC: S-1/Cisplatin, PC: Paclitaxel/Cisplatin.

### Relationship between the expression profile each biomarker candidate and the clinicopathological factors

There was no significant difference between the mRNA expression levels of the seven biomarker candidates (*EGFR*, *ZDHHC14*, *CLDN18.2*, *TIMP1*, *DSG2*, *MUC2*, and *RRM1*) and the clinicopathological factors (*n* = 46) (Supplementary Table 2).

As a reference, the relationship between the expression levels of seven biomarker candidates and the clinicopathological factors in different cohort of locally advanced GC (*n* = 253) was examined, a significant association existed between the expression levels of *DSG* and histological type and tumor depth, of *CLDN18.2* and the incidence of venous invasion, of *EGFR* and that of lymph node metastasis and venous invasion, of *MUC2* and that of venous invasion (Supplementary Table 3).

### Relationship between protein and gene expression determined by immunohistochemical and mRNA expression analyses, respectively, of the same specimens

**Figure 2:**
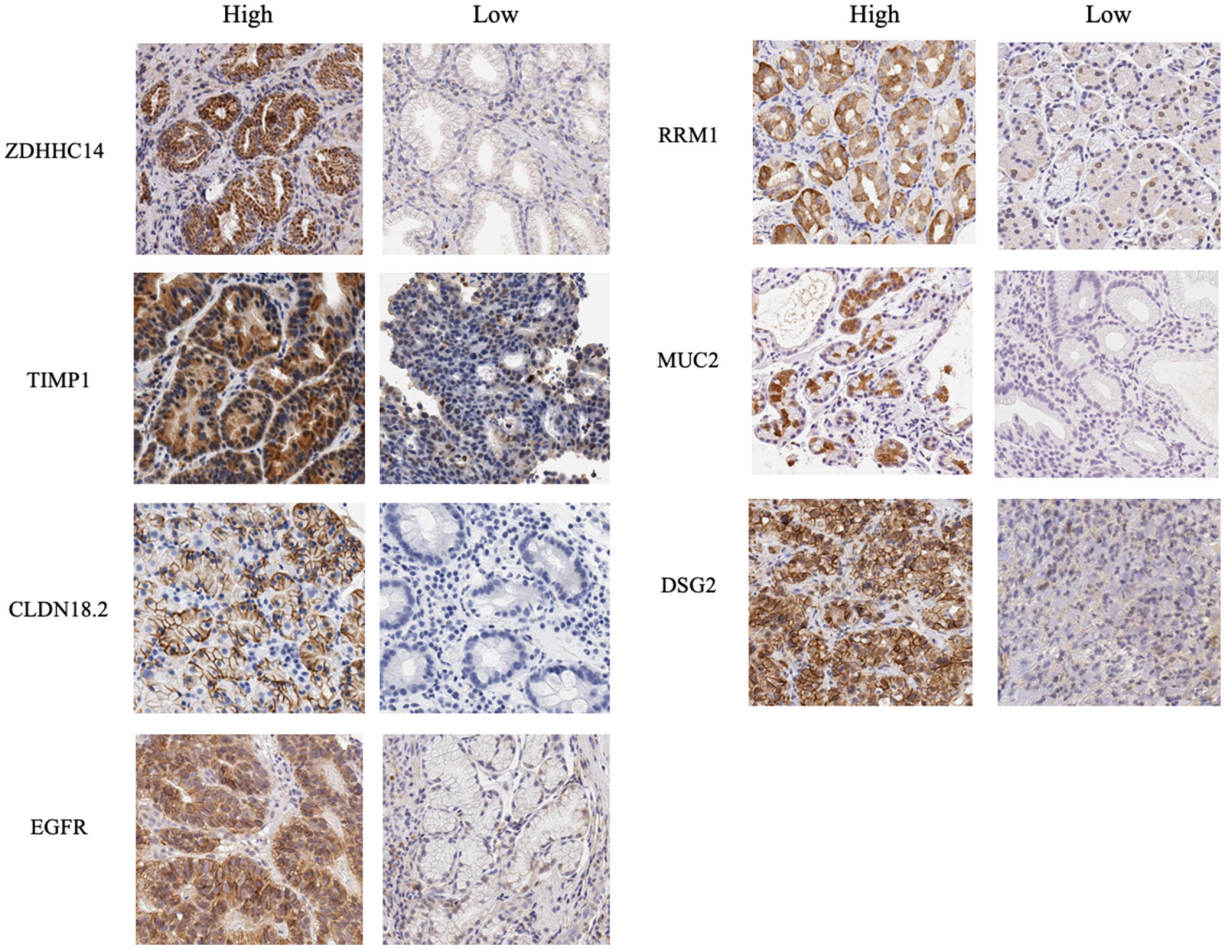
Immunohistochemical images for proteins encoded by seven biomarker candidate genes. Immunohistochemical studies were performed for proteins encoded by seven biomarker candidate genes, ZDHHC14, TIMP1, CLDN18.2, EGFR, RRM1, MUC2 and DSG2 using formalin-fixed, paraffin-embedded biopsy specimens obtained from 24 patients. Immunohistochemical evaluation was performed based on the positive immunostained tumor cells with maximum intensity and the percentage of positive immunostained tumor cells. Representative images of high and low immunostaining of each biomarker candidate are shown.

Representative images of high and low immunostaining for determining the levels of EGFR, ZDHHC14, CLDN18.2, TIMP1, DSG2, MUC2, and RRM1 are shown in Figure 2.

**Figure 3:**
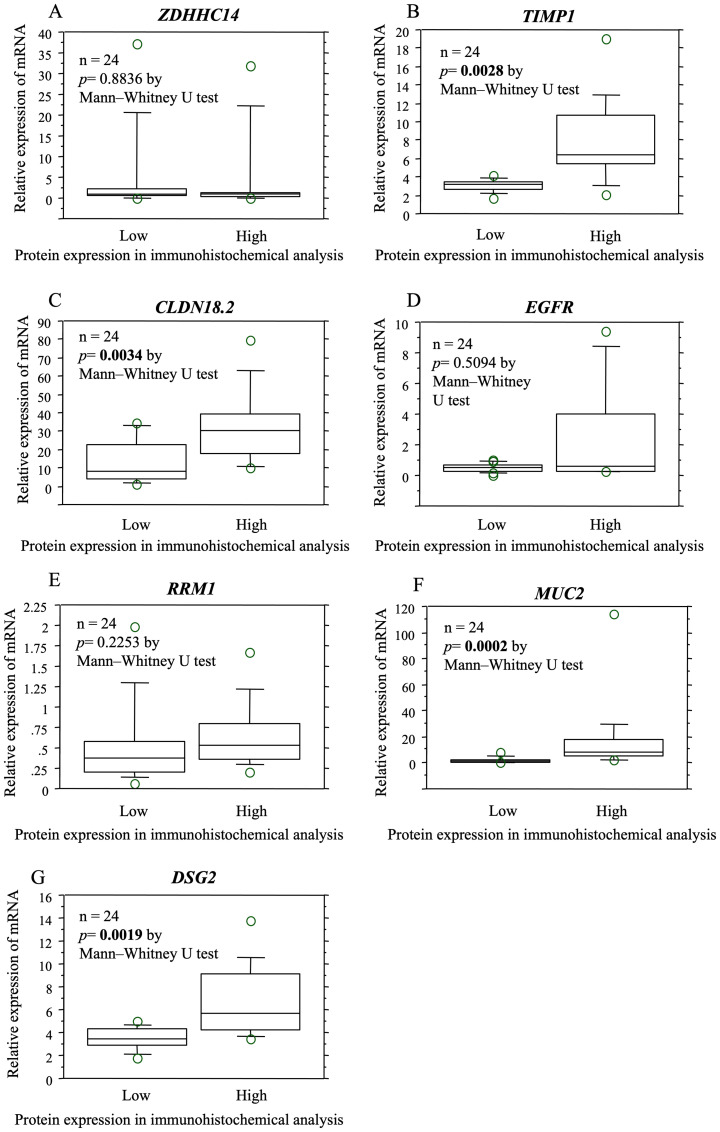
The relationship between protein and gene expression determined by immunohistochemical and mRNA expression analysis in the same specimens. The expression of proteins encoded by the biomarker candidates was examined at the mRNA level in the same samples. Based on the comparison of the mRNA expression levels of samples that were high immunoreactivity and low/negative immunoreactivity, significant differences were observed in the mRNA expression levels of *TIMP1* (**B**), *CLDN18.2* (**C**), and *DSG2* (**G**). There was no significant difference in the expression levels of *ZDHHC14* (**A**), *EGFR* (**D**), *RRM1* (**E**), *MUC2* (**F**).

The expression of proteins encoded by the biomarker candidates was evaluated at the mRNA level in the same samples. Comparison of the mRNA expression levels of samples with high and low immunostaining revealed significant differences between the levels of *TIMP1*, *CLDN18.2*, and *DSG2* (Figure 3).

In addition, for the expression levels of *TIMP1* and *DSG2*, the concordance rates between the mRNA expression levels that were divided into two by the cutoff value and the protein expression levels determined by immunohistochemical analysis were more than 70%. (Table 4). A retrospective study revealed that the pathological response rate of selecting PC when TIMP1 immunostaining was high and selecting SC when TIMP1 immunostaining was low/negative was 80.0%, and the pathological response rate of selecting SC when DSG2 immunostaining was high and selecting PC when DSG2 immunostaining was low was 88.9% (Table 5).

**Table 4:** The relationship between the relative expression of mRNA and proteins in immunohistochemical analysis

**ZDHHC14**			**TIMP1**			**CLDN18.2**		
	IHC High	IHC Low		IHC High	IHC Low		IHC High	IHC Low
mRNA High	5	12	mRNA High	6	3	mRNA High	4	0
mRNA Low	3	4	mRNA Low	4	11	mRNA Low	10	10
Concordance rate: 37.5%			Concordance rate: **70.8%**			Concordance rate: 58.3%		
**RRM1**			**MUC2**			**DSG2**		
	IHC High	IHC Low		IHC High	IHC Low		IHC High	IHC Low
mRNA High	3	2	mRNA High	4	3	mRNA High	9	3
mRNA Low	7	12	mRNA Low	9	6	mRNA Low	4	8
Concordance rate: 62.5%			Concordance rate: 41.7%			Concordance rate: **70.8%**		
**EGFR**								
	IHC High	IHC Low						
mRNA High	4	6						
mRNA Low	2	12						
Concordance rate: 66.7%								

Abbreviation; IHC: immunohistochemistry.

**Table 5:** Pathological response when regimen is selected based on TIMP1 or DSG2 expression on IHC by retrospective analysis

Sample No.	NAC regimen actually administrated	Regimen selected based on TIMP1 expression	Pathological response	NAC regimen actually administrated	Regimen selected based on DSG2 expression	Pathological response
1	***PC***	***PC***	**positive**	PC	SC	
2	PC	SC		PC	SC	
3	SC	PC		***SC***	***SC***	**positive**
4	***SC***	***SC***	negative	***SC***	***SC***	negative
5	PC	SC		PC	SC	
6	***PC***	***PC***	**positive**	***PC***	***PC***	**positive**
7	PC	SC		PC	SC	
8	SC	PC		SC	PC	
9	***SC***	***SC***	**positive**	SC	PC	
10	PC	SC		PC	SC	
11	PC	SC		PC	SC	
12	***PC***	***PC***	**positive**	PC	SC	
13	***SC***	***SC***	**positive**	SC	PC	
14	PC	SC		***PC***	***PC***	**positive**
15	SC	PC		SC	PC	
16	SC	PC		SC	PC	
17	***SC***	***SC***	**positive**	***SC***	***SC***	**positive**
18	PC	SC		PC	SC	
19	***SC***	***SC***	negative	SC	PC	
20	***SC***	***SC***	**positive**	***SC***	***SC***	**positive**
21	PC	SC		***PC***	***PC***	**positive**
22	SC	PC		***SC***	***SC***	**positive**
23	***PC***	***PC***	**positive**	***PC***	***PC***	**positive**
24	SC	PC		SC	PC	
	Pathological response rate when regimen is selected by TIMP1 expression		**80% (8/10)**	Pathological response rate when regimen is selected by DSG2 expression		**88.9% (8/9)**

Abbreviations; NAC: neoadjuvant chemotherapy, IHC: immunohistochemistry, SC: S-1/cisplatin, PC: paclitaxel/cisplatin.

Based on these results, *TIMP1* and *DSG2* were identified as the biomarkers that could possibly predict the pathological response of locally advanced GC to each NAC regimen.

## DISCUSSION

To the best of our knowledge, this is the first study to investigate biomarkers predicting the pathological response to NAC regimens in advanced GC. The findings of the randomized phase II NAC study (COMPASS) suggested that the selection of the NAC regimen and courses did not impact the pathological response. However, it was noteworthy that a pathological complete response was achieved in 10% of the patients who received four courses of either SC or PC. This result suggested that there may be optimal NAC regimens for the treatment of various tumors. Therefore, we hypothesized that if the types of tumors that are likely to elicit a remarkable pathological response to NAC could be identified prior to treatment initiation, further improvements in outcomes could be expected. This study primarily aimed to determine the feasibility of personalizing a NAC regimen to treat locally advanced GC using regimen selection biomarkers.

In this biomarker study, which included the findings of the COMPASS phase II trial [12], the association between the expression levels of 127 preselected genes in the endoscopic biopsy specimens collected before NAC and the pathological response to the SC or PC regimens of NAC were analyzed in patients with locally advanced GC. Based on the mRNA expression levels, *TIMP1*, *DSG2*, *RRM1*, *MUC2*, *EGFR, ZDHHC14*, and *CLDN18.2* were denoted as the potential biomarker candidates that could predict the pathological response of locally advanced GC to each NAC regimen. The examination of the relationship between the expression of the biomarker genes and proteins using immunohistochemistry suggested that TIMP1 and DSG2 could be effective as predictive biomarkers of the pathological response to each NAC regimen.

TIMP1 is a specific inhibitor of simultaneously expressing matrix metalloproteinase. It controls the matrix metalloproteinase-mediated degradation of extracellular matrix protein, which is an essential step in cancer cell invasion and metastasis. High expression of *TIMP1* was observed in GC and has been associated with recurrence and outcomes [14, 15]. With respect to the relation between *TIMP1* and chemotherapy, high *TIMP1* expression correlated with a positive response to 5-fluorouracil-based regimens in patients with colorectal cancer [16, 17]. Moreover, another study reported that elevated tumor tissue *TIMP1* levels were significantly associated with a poor response to paclitaxel-based chemotherapy in patients with breast cancer [18].

DSG2 is one of the components of the cell-cell adherence junction. The association of reduced *DSG2* expression with diffuse-type GC and poor prognosis has been reported earlier [19]. Reportedly, the recombinant adenovirus serotype 3-derived protein (JO-1), which triggers the transient opening of intercellular junctions in epithelial tumors by binding to DSG2, enhanced the antitumor activity of several therapeutic monoclonal antibodies in breast cancer, lung cancer, and GC [20]. Moreover, JO-1 enhances the efficacy of 5-fluorouracil and other chemotherapeutic agents and has been shown to play a role in overcoming drug resistance in several models [21].

A retrospective study revealed that the pathological response rate of selecting NAC regimen based on the expression levels of these biomarkers using immunohistochemistry analysis was high. Although the clinical application of this study requires further validation, it may be a first step towards personalized NAC treatment based on biomarkers.

This study has several limitations. First, the sample size was limited; only 46 (55%) patients were examined in this study, which could have increased the alpha error. For clinical applications, we opine that a validation study in other cohorts with a large sample size and a single blind study are necessary. Second, the tumor heterogeneity poses a challenge. Although biopsy samples for the biomarker analysis were obtained from four to six tumor sites in each patient, it is difficult to evaluate whether the biopsy samples faithfully represented the characteristics of the entire tumor.

In conclusion, the biomarkers predicting the pathological response of locally advanced GC to each NAC regimen were identified. Based on our results, the possibility of personalizing NAC treatment using biomarkers was suggested. Our results might pave the way for clinical trials on biomarker-oriented NAC.

## MATERIALS AND METHODS

### The COMPASS study

The COMPASS study (UMIN-000002595) enrolled patients with clinical stage III GC and with R0 or R1-resectable stage IV GC. Thin-slice CT or multi-detector row CT and diagnostic laparoscopy were mandatory for clinical staging. The T and N criteria were determined precisely based on the protocol. Eligible patients were registered and subsequently randomized by centralized dynamic randomization with the following stratification factors: macroscopic type, esophageal invasion, M1 stage, and creatinine clearance. Patients were randomly assigned to receive NAC with SC or PC. In the SC regimen, S-1 was administered twice daily at a total dose of 80 mg/m^2^ for the first three weeks of a four-week cycle, and cisplatin was administered as an intravenous infusion of 60 mg/m^2^ on day 8 of each cycle, as described previously [22]. In the PC regimen, paclitaxel (60 mg/m^2^) and cisplatin (25 mg/m^2^) were administered on days 1, 8, and 15 in one course; this was repeated every four weeks. The details of NAC treatments have been reported previously [23]. Patients proceeded to surgery after receiving NAC. Standard D2 gastrectomy was performed with the goal of ensuring an R0 resection [24].

The pathological response was evaluated according to the MD Anderson Cancer Centre regression grading systems. Surgical specimens were pathologically categorized as “I” when there was less than 10% or no tumor cell destruction, as “IIa” when there was 10 to 50% destruction of tumor cells, as “IIb” when there was 51 to 90% destruction of tumor cells, “III” when there were viable tumor cells, and “IV” when there was no viable residual tumor (complete response). The pathological responders were patients with tumors eliciting a IIb-, III-, or IV-type response. The protocol of this biomarker study was approved by the institutional review board/ethics committee of each participating institution.

### RNA extraction and complementary DNA (cDNA) synthesis

The present study involved the retrospective collection of formalin-fixed, paraffin-embedded GC tissue specimens that had been obtained endoscopically prior to NAC. The tissue specimens were thinly sliced (thickness, 10 μm); five slices were mounted on glass slides. The cancer site was manually dissected and the sample was transferred to a micro-tube. Total RNA was isolated from GC tissues using NucleoSpin^®^ FFPE RNA XS (MACHEREY-NAGEL GmbH & Co. KG, Düren, Germany). We performed the quality control of RNA under careful observation. We measured OD_260_/OD_280_ to assess the purity of total RNA using a microvolume spectrometer, NanoDrop 2000, (Thermo Fisher Scientific Inc., MA, USA), and measured the total RNA Integrity Number to assess the fragmentation of RNA using an Agilent 2100 Bioanalyzer (Agilent Technologies Inc., Waldbronn, Germany). We prepared cDNA only from samples that met these quality control criteria for RNA. cDNA was synthesized from 0.4 μg of total RNA using an iScript cDNA Synthesis Kit (Bio-Rad Laboratories Ltd., CA, USA). After synthesis, the cDNA was diluted to 0.2 μg/μl with water and stored at -20°C until use.

### Quantitative real-time polymerase chain reaction (PCR)

Quantitative real-time PCR was performed using the iQ SYBR Green Supermix (Bio-Rad Laboratories Ltd, CA, USA). PCR reactions were conducted in a total volume of 15 μl, which included 0.2 μg of cDNA, 0.4 μM of each primer, 7.5 μl of the iQ SYBR Green Supermix containing dATP, dCTP, dGTP, and dTTP at 400 μM each, and 50 units/ml of iTag DNA polymerase. The PCR cycle proceeded as follows: 10 min at 95°C, followed by 40 cycles of denaturation of the cDNA for 10 s at 95°C, annealing for 10 s at a temperature suitable for each gene, and primer extension for 20 s at 72°C, followed by holding for 10 min at 72°C. Melting curve analyses were performed to distinguish specific and nonspecific products and the primer dimers. The quantification of each gene was performed in triplicate. A standard curve was constructed for each run by measuring the human control cDNA levels (Clontech Laboratories, Inc., CA, USA) at three points to evaluate specific mRNA expression in samples. The concentration in each sample was calculated based on its corresponding point of intersection with the standard curve and was then normalized to that of the reference gene, β-actin.

### Gene selection

In this study, the expression levels of 127 genes were measured (Table 6). We first selected 74 genes based on findings from DNA microarray experiments. Next, based on the evidence from current literature, the genes were selected from 13 categories related to tumor progression or survival in GC patients, and 53 genes that did not overlap with the above 74 genes were added.

**Table 6:** Genes investigated (127 genes)

**1. Genes related to the metabolism or activation of anticancer agents**							
*TYMS*	*DPYD*	*UMPS*	*TK1*	*TYMP*	*GGH*	*DUT*	*MTHFR*
*RRM1*	*RRM2*	*FPGS*	*DHFR*	*ERCC1*	*TOP2A*	*MAPT*	*TOP1*
**2. Genes related to growth factors and receptor tyrosine kinases**							
*EGF*	*AREG*	*EREG*	*VEGFA*	*IGF2*	*HGF*	*MET*	*FGFR2*
*EGFR*	*ERBB2*	*KDR*	*IGF1R*	*PDGFRB*			
**3. Genes related to the p13K-AKT, RAS, and RAP1 signaling pathways**							
*PTEN*	*ITGB3*	*PLA2G2A*	*THBS1*				
**4. Tumor suppressor genes**							
*SEMA3B*	*RUNX3*	*MLH1*	*APC*	*DAPK1*	*MGMT*	*CDKN2A*	
**5. Genes related to apoptosis**							
*E2F1*	*BCL2*	*GADD45*	*FAS*	*BIRC5*	*BCL2L11*	*BAX*	*CCND1*
**6. Genes related to cancer stem cells**							
*LGR5*	*PROM1*	*CD44*	*NANOG*	*MSI1*			
**7. Genes related to anticancer drug resistance**							
*ABCG2*	*ABCB1*	*ABCC1*	*CAV1*				
**8. Genes encoding members of the MMP family**							
*MMP2*	*MMP7*	*MMP9*	*MMP11*	*MMP14*	*TIMP1*		
**9. Genes encoding cell adhesion factors and extracellular matrix**							
*CDH17*	*LGALS4*	*VCAM1*	*HPSE*	*DSG2*	*CDX2*		
**10. Genes of the claudin family**							
*CLDN3*	*CLDN4*	*CLDN7*	*CLDN18.2*				
**11. Genes encoding chemokine receptors**							
*CCR7*	*CXCR4*						
**12. Epigenetic repression genes**							
*HDAC1*	*EZH2*						
**13. Genes identified by SAGE and CAST methods**							
*APOE*	*REG4*	*MIA*	*OLFM4*	*SEC11A*	*TSPAN8*	*TM9SF3*	*ZDHHC14*
*CAND1*							
**14. Others**							
*INHBA*	*CEMIP*	*SATB2*	*TNS4*	*HOXB9*	*COL1A2*	*IGF2BP3*	*GDF15*
*CSAG2*	*LAPTM4B*	*SLCO1B3*	*CEACAM6*	*VSNL1*	*MUC12*	*SRPX2*	*SEMA5A*
*TKTL1*	*CCNE1*	*SLPI*	*ESM1*	*PDCD5*	*SLC34A2*	*SULF1*	*CEACAM7*
*SPARC*	*PECAM1*	*IGFBP3*	*ANGPT2*	*MUC2*	*PPARG*	*ESR1*	*PER2*
*ARNTL*	*SIRT1*	*GSTO1*	*GZMA*	*LDHA*	*PTGS2*	*PLAU*	*TGFA*

We first performed hematoxylin-eosin staining and CDH17 and LGALS4 immunostaining using 400 frozen specimens excised by surgery for locally advanced GC and selected the specimens that could be clearly categorized as intestinal or diffused based on histopathological characteristics. Among them, we selected the specimens representing pStage IIIC in which NAC was considered to be most appropriate and which had early recurrence despite receiving adjuvant chemotherapy. Next, total RNA was extracted from the cancer tissue and normal gastric mucosa of the specimens, and the ratio of expression of the 28,869 genes in cancer tissue and normal gastric mucosa was calculated using DNA microarray analysis. From the 267 genes that were expressed four times or more than four times the expression ratio in cancer tissues/normal gastric mucosa, we selected 74 genes that have been associated with cancer in relevant literature. In addition, we searched for genes from 13 categories related to tumor progression or survival in patients with GC, based on evidence from current literature. The 13 categories are: genes related to metabolism or activation of anticancer agents, genes related to growth factor and receptor tyrosine kinases, genes related to the p13K-AKT, RAS and RAP1 signaling pathways, tumor suppressor genes, genes related to apoptosis, genes related to cancer stem cells, genes related to anticancer drug resistance, genes encoding members of MMP family, genes encoding cell adhesion factors and extracellular matrix, genes of the claudin family, genes encoding chemokine receptors, epigenetic regression genes, genes identified by serial analysis of gene expression (SAGE) or the Escherichia coli ampicillin secretion trap (CAST) method [25]. The total number of genes included were 127, except for those that were duplicated.

### Immunohistochemical analyses of proteins encoded by the biomarker candidate genes

Immunohistochemical analyses were performed for EGFR, ZDHHC14, CLDN18.2, TIMP1, DSG2, MUC2, and RRM1 using formalin-fixed, paraffin-embedded biopsy specimens obtained from 24 patients. The tissue sections were deparaffinized and soaked in 10 mM sodium citrate buffer (pH 6.0) at 121°C for 15 min to retrieve the cellular antigens. After blocking, the sections were incubated overnight with the primary antibodies at 4°C to allow antigen-antibody reactions to occur. The anti-EGFR antibody (ab52894, Abcam PLC, Cambridge, UK), anti-ZDHHC14 antibody (ab237503, Abcam), anti-CLDN18.2 antibody (ab225512, Abcam), anti-TIMP1 antibody (ab109125, Abcam), anti-DSG2 antibody (ab150372, Abcam), and anti-RRM1 antibody (ab137114, Abcam) were used as primary antibodies. Preliminary examination was performed using positive controls to determine the optimal dilution of each antibody and antigen-antibody reactions were performed subsequently. A peroxidase-labeled polymer (EnVision+, rabbit, DAKO, Glostrup, Denmark) was used to detect the signals from the antigen-antibody reactions. All sections were counterstained with hematoxylin. Immunohistochemical evaluation was performed according to a modified immunoreactivity scoring system (IRS): Category A categorized the positive immunostained tumor cells with maximum intensity as absent (0), weak (1), moderate (2), and strong (3). Category B categorized the percentage of positive immunostained tumor cells into four grades (0, 1, 2, 3) based on the marker-specific approach. The scores from categories A and B added up to an IRS of 0 to 6. The IRS of 0 to 4 was defined as low/negative immunoreactivity and those of 5 to 6 was defined as high immunoreactivity.

The relationship between protein and gene expression determined by immunohistochemical and mRNA expression analysis in the same specimens was then examined. In addition, the concordance rate between the mRNA expression levels which were divided into two by the cutoff value and the protein expression levels determined by immunohistochemical analysis was examined.

### Statistical analysis

In the identification of biomarker candidates at the mRNA level that predict the pathological response to each NAC regimen, we formed two subgroups by altering the cutoff values of the expression levels of each of the 127 genes and searched for cutoff values that were associated with the interaction having minimum *P*-values between the two subgroups and the pathological response to either SC or PC in each gene using logistic regression analysis. Next, we identified the genes with statistically significant interactions (*P* < 0.01). In our analysis of the relationship between protein and gene expression using immunohistochemical and mRNA expression analyses, respectively, the Mann–Whitney *U* test was used for comparing the averages of mRNA expression levels.

## SUPPLEMENTARY MATERIALS







## Abbreviations

GC: gastric cancer
NAC: neoadjuvant chemotherapy
SC: S-1/cisplatin
PC: S-1/paclitaxel
OS: overall survival
P0CY1: no peritoneal metastasis and peritoneal lavage cytology positive
JO-1: the recombinant adenovirus serotype 3-derived protein
PCR: polymerase chain reaction
cDNA: complementary DNA
