# Supplementary material for: Biomarker analysis to predict the pathological response to neoadjuvant chemotherapy in locally advanced gastric cancer: An exploratory biomarker study of COMPASS, a randomized phase II trial
Source: Oncotarget. 2020 Jul 28;11(30):2906–18. doi: 10.18632/oncotarget.27658 (PMC7392622; doi:10.18632/oncotarget.27658)
Supplement: Supplementary file 1 [file oncotarget-11-2906-s001.pdf]

Biomarker analysis to predict the pathological response to neoadjuvant chemotherapy in locally advanced gastric cancer: An exploratory biomarker study of COMPASS, a randomized phase II trial

SUPPLEMENTARY MATERIALS

Supplementary Table 1: The relation between the expression levels of 127 genes and pathological response to either SC or PC. See Supplementary Table 1

Supplementary Table 2: Relation between mRNA expression and clinicopathological features (biomarker analysis cohort, *n* = 46)

	ZDHHC14			*TIMP1*			*CLDN18.2*		
**Variables/categories**	**≧0.608 (*n* = 31)**	**< 0.608 (*n* = 15)**	***P*-value**	**≧10.473 (*n* = 8)**	**≧10.473 (*n* = 38)**	***P*-value**	**≧23.564 (*n* = 18)**	**< 23.564 (*n* = 28)**	***P*-value**
Age (years)									
< 67	20	8	0.4463	2	26	0.0022	9	9	0.2258
≧67	11	7		6	12		9	19	
Gender									
Male	22	11	0.8673	1	11	0.3356	13	21	0.8341
Female	9	4		7	27		5	7	
Histologic type									
Differentiated	13	5	0.5752	3	15	0.9172	7	11	0.9785
Undifferentiated	18	10		5	23		11	17	

	EGFR			*RRM1*			*MUC2*		
**Variables/categories**	**≧ 0.549 (*n* = 21)**	**< 0.549 (*n* = 25)**	***P*-value**	**≧ 0.803 (*n* = 12)**	**< 0.803 (*n* = 34)**	***P*-value**	**≧14.04 (*n* = 9)**	**≧14.04 (*n* = 37)**	***P*-value**
Age (years)									
< 67	13	15	0.8951	5	23	0.1129	4	24	0.2603
≧67	8	10		7	11		5	13	
Gender									
Male	16	18	0.7472	8	26	0.5061	5	29	0.162
Female	5	7		4	8		4	8	
Histologic type									
Differentiated	9	9	0.9172	6	12	0.3695	3	15	0.6911
Undifferentiated	12	16		6	22		6	22	

	DSG2		
**Variables/categories**	**≧ 4.312 (*n* = 24)**	**< 4.312 (*n* = 22)**	***P*-value**
Age (years)			
< 67	9	19	0.2258
≧67	9	9	
Gender			
Male	13	22	0.6222
Female	5	6	
Histologic type			
Differentiated	7	11	0.9785
Undifferentiated	11	17	

Supplementary Table 3: Relation between mRNA expression and clinicopathological features in pStage II/III gastric cancer (different cohort, *n* = 253). See Supplementary Table 3
