# Supplementary material for: Biomarker analysis to predict the pathological response to neoadjuvant chemotherapy in locally advanced gastric cancer: An exploratory biomarker study of COMPASS, a randomized phase II trial
Source: Oncotarget. 2020 Jul 28;11(30):2906–18. doi: 10.18632/oncotarget.27658 (PMC7392622; doi:10.18632/oncotarget.27658)
Supplement: Supplementary file 2 [file oncotarget-11-2906-s002.docx]

| **Supplementary Table 1: The relation between the expression levels of 127 genes and pathological response to either SC or PC** | | | | | | | | | |
| --- | --- | --- | --- | --- | --- | --- | --- | --- | --- |
| **Biomarker** | **Category** | **S-1/cisplatin**  **arm** | | | **Paclitaxel/cisplatin arm** | | | **Comparison between the treatment groups** | ***P*-value for treatment interaction** |
|  |  | **No.** | **Responders** | | **No.** | **Responders** | |  |  |
|  |  |  | **No.** | **%** |  | **No.** | **%** | ***P*-value** |  |
| ***ZDHHC14*** | ＜0.608 | 8 | 7 | 87.5 | 7 | 1 | 14.3 | 0.0101 | **0.0002** |
|  | ≧0.608 | 15 | 5 | 33.3 | 16 | 12 | 75.0 | 0.0319 |  |
| ***TIMP1*** | ＜10.473 | 18 | 7 | 38.9 | 20 | 13 | 65.0 | 0.1927 | **0.0013** |
|  | ≧10.473 | 5 | 5 | 100 | 3 | 0 | 0.0 | 0.0179 |  |
| ***CLDN18.2*** | ＜23.564 | 11 | 8 | 72.7 | 17 | 7 | 41.2 | 0.1367 | **0.0016** |
|  | ≧23.564 | 12 | 4 | 33.3 | 6 | 6 | 100 | 0.0128 |  |
| ***EGFR*** | ＜0.549 | 13 | 9 | 69.2 | 12 | 4 | 33.3 | 0.1152 | **0.0028** |
|  | ≧0.549 | 10 | 3 | 30.0 | 11 | 9 | 81.8 | 0.0299 |  |
| ***RRM1*** | ＜0.803 | 15 | 7 | 46.7 | 18 | 13 | 72.2 | 0.1686 | **0.0075** |
|  | ≧0.803 | 8 | 5 | 62.5 | 5 | 0 | 0.0 | 0.0754 |  |
| ***MUC2*** | ＜14.04 | 18 | 7 | 38.9 | 19 | 12 | 63.2 | 0.1939 | **0.0077** |
|  | ≧14.04 | 5 | 5 | 100 | 4 | 1 | 25.0 | 0.0476 |  |
| ***DSG2*** | ＜4.312 | 10 | 3 | 30.0 | 12 | 9 | 75.0 | 0.0836 | **0.0091** |
|  | ≧4.312 | 13 | 9 | 69.2 | 11 | 4 | 36.7 | 0.2173 |  |
| *MIA* | ＜0.042 | 4 | 4 | 100 | 12 | 5 | 41.7 | 0.0885 | 0.0106 |
|  | ≧0.042 | 19 | 8 | 42.1 | 11 | 8 | 72.7 | 0.1424 |  |
| *CAV1* | ＜0.2295 | 4 | 4 | 100 | 12 | 5 | 41.7 | 0.0885 | 0.0106 |
|  | ≧0.2295 | 19 | 8 | 42.1 | 11 | 8 | 72.7 | 0.1424 |  |
| *CDX2* | ＜5.324 | 6 | 1 | 16.7 | 3 | 3 | 100 | 0.0476 | 0.0124 |
|  | ≧5.324 | 17 | 11 | 64.7 | 20 | 10 | 50.0 | 0.5085 |  |
| *KI-67* | ＜0.922 | 18 | 8 | 44.4 | 20 | 13 | 65.0 | 0.3275 | 0.0126 |
|  | ≧0.922 | 5 | 4 | 80.0 | 3 | 0 | 0.0 | 0.1429 |  |
| *CDH17* | ＜9.354 | 16 | 6 | 37.5 | 19 | 12 | 63.2 | 0.1811 | 0.0159 |
|  | ≧9.354 | 7 | 6 | 85.7 | 4 | 1 | 25.0 | 0.0879 |  |
| *BCRP* | ＜1.446 | 16 | 6 | 37.5 | 17 | 11 | 64.7 | 0.1694 | 0.0161 |
|  | ≧1.446 | 7 | 6 | 85.7 | 6 | 2 | 33.3 | 0.1026 |  |
| *MMP11* | ＜0.31 | 10 | 5 | 50.0 | 13 | 11 | 84.6 | 0.1688 | 0.0171 |
|  | ≧0.31 | 13 | 7 | 53.8 | 10 | 2 | 20.0 | 0.1968 |  |
| *PLA2G2A* | ＜1.837 | 14 | 4 | 28.6 | 15 | 9 | 60.0 | 0.1394 | 0.0191 |
|  | ≧1.837 | 9 | 8 | 88.9 | 8 | 4 | 50.0 | 0.1312 |  |
| *TP* | ＜35.968 | 12 | 8 | 66.7 | 16 | 7 | 43.8 | 0.2761 | 0.0208 |
|  | ≧35.968 | 11 | 4 | 36.4 | 7 | 6 | 85.7 | 0.0656 |  |
| *Bmal1* | ＜0.251 | 3 | 3 | 100 | 12 | 5 | 41.7 | 0.2 | 0.0232 |
|  | ≧0.251 | 20 | 9 | 45.0 | 11 | 8 | 72.7 | 0.2581 |  |
| *EREG* | ＜0.069 | 17 | 7 | 41.2 | 13 | 9 | 69.2 | 0.1589 | 0.027 |
|  | ≧0.069 | 6 | 5 | 83.3 | 10 | 4 | 40.0 | 0.1451 |  |
| *FAS* | ＜0.862 | 19 | 9 | 47.4 | 20 | 13 | 65.0 | 0.3406 | 0.0272 |
|  | ≧0.862 | 4 | 3 | 75.0 | 3 | 0 | 0.0 | 0.1429 |  |
| *PTGS2* | ＜0.232 | 4 | 3 | 75.0 | 12 | 4 | 33.3 | 0.2615 | 0.0276 |
|  | ≧0.232 | 19 | 9 | 47.4 | 11 | 9 | 81.8 | 0.1213 |  |
| *CEACAM7* | ＜5.527 | 13 | 5 | 38.5 | 9 | 7 | 77.8 | 0.0991 | 0.0295 |
|  | ≧5.527 | 10 | 7 | 70.0 | 14 | 6 | 42.9 | 0.2397 |  |
| *CLDN7* | ＜11.076 | 17 | 7 | 41.2 | 17 | 11 | 64.7 | 0.3028 | 0.0315 |
|  | ≧11.076 | 6 | 5 | 83.3 | 6 | 2 | 33.3 | 0.2424 |  |
| *SULF1* | ＜3.037 | 12 | 6 | 50.0 | 16 | 12 | 75.0 | 0.2425 | 0.0321 |
|  | ≧3.037 | 11 | 6 | 54.5 | 7 | 1 | 14.3 | 0.1507 |  |
| *EZH2* | ＜3.472 | 18 | 9 | 50.0 | 19 | 13 | 68.4 | 0.3245 | 0.0355 |
|  | ≧3.472 | 5 | 3 | 60.0 | 4 | 0 | 0.0 | 0.1667 |  |
| *PECAM1* | ＜5.043 | 13 | 8 | 61.5 | 13 | 5 | 38.5 | 0.4338 | 0.0368 |
|  | ≧5.043 | 10 | 4 | 40.0 | 10 | 8 | 80.0 | 0.1698 |  |
| *GZMA* | ＜1.246 | 11 | 6 | 54.5 | 11 | 3 | 27.3 | 0.3869 | 0.0373 |
|  | ≧1.246 | 12 | 6 | 50.0 | 12 | 10 | 83.3 | 0.193 |  |
| *COL1A2* | ＜216.5 | 16 | 7 | 43.8 | 21 | 13 | 61.9 | 0.3309 | 0.0434 |
|  | ≧216.5 | 7 | 5 | 71.4 | 2 | 0 | 0.0 | 0.1667 |  |
| *APOE* | ＜1.2195 | 5 | 4 | 80.0 | 12 | 5 | 41.7 | 0.2941 | 0.0469 |
|  | ≧1.2195 | 18 | 8 | 44.4 | 11 | 8 | 72.7 | 0.249 |  |
| *DAPK1* | ＜0.138 | 8 | 2 | 25.0 | 14 | 9 | 64.3 | 0.1827 | 0.0484 |
|  | ≧0.138 | 15 | 10 | 66.7 | 9 | 4 | 44.4 | 0.4028 |  |
| *SLC34A2* | ＜0.306 | 4 | 3 | 75.0 | 5 | 1 | 20.0 | 0.2063 | 0.0509 |
|  | ≧0.306 | 19 | 9 | 47.4 | 18 | 12 | 66.7 | 0.3245 |  |
| *CEACAM6* | ＜4.872 | 10 | 3 | 30.0 | 7 | 5 | 71.4 | 0.1534 | 0.0541 |
|  | ≧4.872 | 13 | 9 | 69.2 | 16 | 8 | 50.0 | 0.4515 |  |
| *MRP1* | ＜7.225 | 18 | 8 | 44.4 | 19 | 12 | 63.2 | 0.3299 | 0.0541 |
|  | ≧7.225 | 5 | 4 | 80.0 | 4 | 1 | 25.0 | 0.2063 |  |
| *CXCR4* | ＜4.698 | 20 | 11 | 55.0 | 18 | 8 | 44.4 | 0.7459 | 0.055 |
|  | ≧4.698 | 3 | 1 | 33.3 | 5 | 5 | 100 | 0.1071 |  |
| *ERCC1* | ＜4.102 | 21 | 10 | 47.6 | 19 | 12 | 63.2 | 0.3596 | 0.0552 |
|  | ≧4.102 | 2 | 2 | 100 | 4 | 1 | 25.0 | 0.4 |  |
| *AREG* | ＜1.997 | 21 | 10 | 47.6 | 19 | 12 | 63.2 | 0.3596 | 0.0552 |
|  | ≧1.997 | 2 | 2 | 100 | 4 | 1 | 25.0 | 0.4 |  |
| *TNS4* | ＜0.57 | 19 | 8 | 42.1 | 15 | 9 | 60.0 | 0.4905 | 0.0571 |
|  | ≧0.57 | 4 | 4 | 100 | 8 | 4 | 50.0 | 0.2081 |  |
| *TS* | ＜2.68 | 19 | 9 | 47.4 | 21 | 13 | 61.9 | 0.5254 | 0.0582 |
|  | ≧2.68 | 4 | 3 | 75.0 | 2 | 0 | 0.0 | 0.4 |  |
| *SATB2* | ＜3.096 | 17 | 8 | 47.1 | 18 | 12 | 66.7 | 0.3145 | 0.0613 |
|  | ≧3.096 | 6 | 4 | 66.7 | 5 | 1 | 20.0 | 0.2424 |  |
| *OATP1B3* | ＜0.434 | 19 | 11 | 57.9 | 17 | 8 | 47.1 | 0.7388 | 0.0632 |
|  | ≧0.434 | 4 | 1 | 25.0 | 6 | 5 | 83.3 | 0.1905 |  |
| *TSPAN8* | ＜15.314 | 18 | 9 | 50.0 | 20 | 13 | 65.0 | 0.5118 | 0.0644 |
|  | ≧15.314 | 5 | 3 | 60.0 | 3 | 0 | 0.0 | 0.1964 |  |
| *MSI1* | ＜0.468 | 6 | 5 | 83.3 | 7 | 3 | 42.9 | 0.2657 | 0.0656 |
|  | ≧0.468 | 17 | 7 | 41.2 | 16 | 10 | 62.5 | 0.3028 |  |
| *IGF2* | ＜0.51 | 5 | 3 | 60.0 | 6 | 1 | 16.7 | 0.2424 | 0.0667 |
|  | ≧0.51 | 18 | 9 | 50.0 | 17 | 12 | 70.9 | 0.3053 |  |
| *REG4* | ＜31.2135 | 15 | 7 | 46.7 | 18 | 12 | 66.7 | 0.3041 | 0.0674 |
|  | ≧31.2135 | 8 | 5 | 62.5 | 5 | 1 | 20 | 0.2657 |  |
| *DPD* | ＜1.854 | 15 | 7 | 46.7 | 18 | 12 | 66.7 | 0.3041 | 0.0674 |
|  | ≧1.854 | 8 | 5 | 62.5 | 5 | 1 | 20.0 | 0.2657 |  |
| *SIRT1* | ＜28.524 | 22 | 12 | 54.5 | 19 | 9 | 47.4 | 0.7579 | 0.0679 |
|  | ≧28.524 | 1 | 0 | 0.0 | 4 | 4 | 100 | 0.2 |  |
| *RUNX3* | ＜1.44 | 17 | 8 | 47.1 | 21 | 13 | 61.9 | 0.5134 | 0.071 |
|  | ≧1.44 | 6 | 4 | 66.7 | 2 | 0 | 0.0 | 0.4286 |  |
| *PTEN* | ＜11.818 | 17 | 10 | 58.8 | 17 | 8 | 47.1 | 0.7319 | 0.0732 |
|  | ≧11.818 | 6 | 2 | 33.3 | 6 | 5 | 83.3 | 0.2424 |  |
| *CLDN3* | ＜4.381 | 10 | 4 | 40.0 | 6 | 5 | 83.3 | 0.1451 | 0.0761 |
|  | ≧4.381 | 13 | 8 | 61.5 | 17 | 8 | 47.1 | 0.4837 |  |
| *PPARG* | ＜5.176 | 11 | 7 | 63.6 | 12 | 5 | 41.7 | 0.4137 | 0.0804 |
|  | ≧5.176 | 12 | 5 | 41.7 | 11 | 8 | 72.7 | 0.2138 |  |
| *THBS1* | ＜2.756 | 6 | 2 | 33.3 | 11 | 8 | 72.7 | 0.1618 | 0.0815 |
|  | ≧2.756 | 17 | 10 | 58.8 | 12 | 5 | 41.7 | 0.4622 |  |
| *TRAG3* | ＜1.104 | 10 | 7 | 70.0 | 11 | 5 | 45.5 | 0.387 | 0.0833 |
|  | ≧1.104 | 13 | 5 | 38.5 | 12 | 8 | 66.7 | 0.2377 |  |
| *CCR7* | ＜4.764 | 13 | 8 | 61.5 | 12 | 5 | 41.7 | 0.4338 | 0.0842 |
|  | ≧4.764 | 10 | 4 | 40.0 | 11 | 8 | 72.7 | 0.1984 |  |
| *SPARC* | ＜5.769 | 6 | 5 | 83.3 | 12 | 6 | 50.0 | 0.3156 | 0.0848 |
|  | ≧5.769 | 17 | 7 | 41.2 | 11 | 7 | 63.6 | 0.4401 |  |
| *GGH* | ＜0.155 | 14 | 8 | 57.1 | 16 | 7 | 43.8 | 0.7152 | 0.08578 |
|  | ≧0.155 | 9 | 4 | 44.4 | 7 | 6 | 85.7 | 0.1451 |  |
| *IGF2BP3* | ＜33.851 | 22 | 12 | 54.5 | 20 | 10 | 50.0 | 1 | 0.0869 |
|  | ≧33.851 | 1 | 0 | 0.0 | 3 | 3 | 100 | 0.25 |  |
| *MAPT* | ＜1.62 | 14 | 9 | 64.3 | 11 | 5 | 45.5 | 0.4347 | 0.0896 |
|  | ≧1.62 | 9 | 3 | 33.3 | 12 | 8 | 66.7 | 0.1984 |  |
| *TGFA* | ＜0.134 | 19 | 11 | 57.9 | 21 | 11 | 52.4 | 0.7605 | 0.0898 |
|  | ≧0.134 | 4 | 1 | 25.0 | 2 | 2 | 100 | 0.4 |  |
| *GDF15* | ＜5.4295 | 18 | 10 | 55.6 | 16 | 7 | 43.8 | 0.7319 | 0.0966 |
|  | ≧5.4295 | 5 | 2 | 40.0 | 7 | 6 | 85.7 | 0.2222 |  |
| *TM9SF3* | ＜8.376 | 14 | 8 | 57.1 | 19 | 13 | 68.4 | 0.7157 | 0.1044 |
|  | ≧8.376 | 9 | 4 | 44.4 | 4 | 0 | 0.0 | 0.228 |  |
| *BIRC5* | ＜1.513 | 5 | 4 | 80.0 | 1 | 0 | 0.0 | 0.3333 | 0.1055 |
|  | ≧1.513 | 18 | 8 | 44.4 | 22 | 13 | 59.1 | 0.5254 |  |
| *MMP9* | ＜0.171 | 3 | 1 | 33.3 | 3 | 3 | 100 | 0.4 | 0.111 |
|  | ≧0.171 | 20 | 11 | 55.0 | 20 | 10 | 50.0 | 1 |  |
| *LAPTM4B* | ＜5.975 | 21 | 10 | 47.6 | 18 | 11 | 61.1 | 0.5233 | 0.112 |
|  | ≧5.975 | 2 | 2 | 100 | 5 | 2 | 40.0 | 0.4286 |  |
| *IGFBP3* | ＜42.797 | 2 | 2 | 100 | 3 | 1 | 33.3 | 0.4 | 0.1121 |
|  | ≧42.797 | 21 | 10 | 47.6 | 20 | 12 | 60.0 | 0.5359 |  |
| *VSNL1* | ＜0.4605 | 2 | 2 | 100 | 3 | 1 | 33.3 | 0.4 | 0.11212 |
|  | ≧0.4605 | 21 | 10 | 47.6 | 20 | 12 | 60.0 | 0.5359 |  |
| *APC* | ＜0.122 | 3 | 0 | 0.0 | 5 | 3 | 60.0 | 0.1964 | 0.1134 |
|  | ≧0.122 | 20 | 12 | 60.0 | 18 | 10 | 55.6 | 1 |  |
| *CCNE1* | ＜1.699 | 17 | 9 | 52.9 | 20 | 13 | 65.0 | 0.5164 | 0.1155 |
|  | ≧1.699 | 6 | 3 | 50.0 | 3 | 0 | 0.0 | 0.4643 |  |
| *VEGFA* | ＜6.61 | 1 | 0 | 0.0 | 2 | 2 | 100 | 0.3333 | 0.1162 |
|  | ≧6.61 | 22 | 12 | 54.5 | 21 | 11 | 52.4 | 1 |  |
| *HER2* | ＜0.633 | 18 | 9 | 50.0 | 21 | 13 | 61.9 | 0.5279 | 0.1186 |
|  | ≧0.633 | 5 | 3 | 60.0 | 2 | 0 | 0.0 | 0.4286 |  |
| *CD133* | ＜3.135 | 18 | 9 | 50.0 | 21 | 13 | 61.9 | 0.5279 | 0.1186 |
|  | ≧3.135 | 5 | 3 | 60.0 | 2 | 0 | 0.0 | 0.4286 |  |
| *LDHA* | ＜7.827 | 7 | 4 | 57.1 | 5 | 1 | 20.0 | 0.2929 | 0.1207 |
|  | ≧7.827 | 16 | 8 | 50.0 | 18 | 12 | 66.7 | 0.4867 |  |
| *HOXB9* | ＜12.985 | 19 | 10 | 52.6 | 18 | 8 | 44.4 | 0.7459 | 0.1212 |
|  | ≧12.985 | 4 | 2 | 50.0 | 5 | 5 | 100 | 0.1667 |  |
| *GADD45* | ＜12.14 | 19 | 10 | 52.6 | 18 | 8 | 44.4 | 0.7459 | 0.1212 |
|  | ≧12.14 | 4 | 2 | 50.0 | 5 | 5 | 100 | 0.1667 |  |
| *PDGFR* | ＜0.766 | 7 | 5 | 71.4 | 11 | 5 | 45.5 | 0.3665 | 0.1244 |
|  | ≧0.766 | 16 | 7 | 43.8 | 12 | 8 | 66.7 | 0.2761 |  |
| *MTHFR* | ＜1.146 | 18 | 8 | 44.4 | 20 | 12 | 60.0 | 0.516 | 0.1307 |
|  | ≧1.146 | 5 | 4 | 80.0 | 3 | 1 | 33.3 | 0.4643 |  |
| *ESR1* | ＜0.018 | 19 | 11 | 57.9 | 16 | 8 | 50.0 | 0.7397 | 0.1405 |
|  | ≧0.018 | 4 | 1 | 25.0 | 7 | 5 | 71.4 | 0.2424 |  |
| *HGF* | ＜2.098 | 10 | 6 | 60.0 | 12 | 10 | 83.3 | 0.3476 | 0.1412 |
|  | ≧2.098 | 13 | 6 | 46.2 | 11 | 3 | 27.3 | 0.4226 |  |
| *SLPI* | ＜4.686 | 6 | 4 | 66.7 | 8 | 3 | 37.5 | 0.5921 | 0.1435 |
|  | ≧4.686 | 17 | 8 | 47.1 | 15 | 10 | 66.7 | 0.3075 |  |
| *MMP7* | ＜9.529 | 15 | 8 | 53.3 | 13 | 5 | 38.5 | 0.4757 | 0.1451 |
|  | ≧9.529 | 8 | 4 | 50.0 | 10 | 8 | 80.0 | 0.3213 |  |
| *PER2* | ＜1.716 | 18 | 10 | 55.6 | 17 | 8 | 47.1 | 0.7395 | 0.1464 |
|  | ≧1.716 | 5 | 2 | 40.0 | 6 | 5 | 83.3 | 0.2424 |  |
| *CD44* | ＜1.176 | 5 | 2 | 40.0 | 6 | 5 | 83.3 | 0.2424 | 0.1464 |
|  | ≧1.176 | 18 | 10 | 55.6 | 17 | 8 | 47.1 | 0.7395 |  |
| *MMP14* | ＜29.109 | 3 | 2 | 66.7 | 7 | 2 | 28.6 | 0.5 | 0.1465 |
|  | ≧29.109 | 20 | 10 | 50.0 | 16 | 11 | 68.8 | 0.3204 |  |
| *MET* | ＜13.088 | 20 | 11 | 55.0 | 17 | 8 | 47.1 | 0.7459 | 0.1484 |
|  | ≧13.088 | 3 | 1 | 33.3 | 6 | 5 | 83.3 | 0.2262 |  |
| *MUC12* | ＜0.374 | 16 | 7 | 43.8 | 16 | 10 | 62.5 | 0.4795 | 0.1534 |
|  | ≧0.374 | 7 | 5 | 71.4 | 7 | 3 | 42.9 | 0.5921 |  |
| *IGF1R* | ＜0.453 | 5 | 4 | 80.0 | 10 | 5 | 50.0 | 0.5804 | 0.1657 |
|  | ≧0.453 | 18 | 8 | 44.4 | 13 | 8 | 61.5 | 0.4725 |  |
| *TP53* | ＜0.752 | 6 | 3 | 50.0 | 10 | 8 | 80.0 | 0.2995 | 0.1664 |
|  | ≧0.752 | 17 | 9 | 52.9 | 13 | 5 | 38.5 | 0.4837 |  |
| *DUT* | ＜13.211 | 9 | 3 | 33.3 | 13 | 8 | 61.5 | 0.387 | 0.1694 |
|  | ≧13.211 | 14 | 9 | 64.3 | 10 | 5 | 50.0 | 0.6785 |  |
| *LGR5* | ＜0.5 | 4 | 1 | 25.0 | 4 | 3 | 75.0 | 0.4857 | 0.1699 |
|  | ≧0.5 | 19 | 11 | 57.9 | 19 | 10 | 52.6 | 1 |  |
| *MLH1* | ＜1.398 | 17 | 8 | 47.1 | 22 | 13 | 59.1 | 0.5279 | 0.1776 |
|  | ≧1.398 | 6 | 4 | 66.6 | 1 | 0 | 0.0 | 0.4288 |  |
| *MMP2* | ＜0.539 | 8 | 3 | 37.5 | 7 | 5 | 71.4 | 0.3147 | 0.1785 |
|  | ≧0.539 | 15 | 9 | 60.0 | 16 | 8 | 50.0 | 0.7224 |  |
| *OLFM4* | ＜6.784 | 5 | 2 | 40.0 | 13 | 9 | 69.2 | 0.326 | 0.1867 |
|  | ≧6.784 | 18 | 10 | 55.6 | 10 | 4 | 40.0 | 0.6946 |  |
| *BCL2* | ＜17.426 | 21 | 11 | 52.4 | 18 | 8 | 44.4 | 0.7512 | 0.1963 |
|  | ≧17.426 | 2 | 1 | 50.0 | 5 | 5 | 100 | 0.2857 |  |
| *GSTO1* | ＜7.785 | 21 | 11 | 52.4 | 18 | 8 | 44.4 | 0.7512 | 0.1963 |
|  | ≧7.785 | 2 | 1 | 50.0 | 5 | 5 | 100 | 0.2857 |  |
| *SRPX2* | ＜4.564 | 20 | 10 | 50.0 | 19 | 12 | 63.2 | 0.5231 | 0.1996 |
|  | ≧4.564 | 3 | 2 | 66.7 | 4 | 1 | 25.0 | 0.4857 |  |
| *INHBA* | ＜1.2495 | 16 | 6 | 37.5 | 21 | 12 | 57.1 | 0.3245 | 0.2036 |
|  | ≧1.2495 | 7 | 6 | 85.7 | 2 | 1 | 50.0 | 0.4167 |  |
| *NANOG* | ＜25.95 | 18 | 10 | 55.6 | 15 | 7 | 46.7 | 0.7319 | 0.2043 |
|  | ≧25.95 | 5 | 2 | 40.0 | 8 | 6 | 75.0 | 0.2929 |  |
| *TOP2A* | ＜0.722 | 21 | 10 | 47.6 | 19 | 11 | 57.9 | 0.545 | 0.2059 |
|  | ≧0.722 | 2 | 2 | 100 | 4 | 2 | 50.0 | 0.4667 |  |
| *MDR1* | ＜3.17 | 19 | 10 | 52.6 | 21 | 13 | 61.9 | 0.7496 | 0.2096 |
|  | ≧3.17 | 4 | 2 | 50.0 | 2 | 0 | 0.0 | 0.4667 |  |
| *PLAU* | ＜0.103 | 17 | 9 | 52.9 | 18 | 12 | 66.7 | 0.4998 | 0.2151 |
|  | ≧0.103 | 6 | 3 | 50.0 | 5 | 1 | 20.0 | 0.5455 |  |
| *FGF2R* | ＜0.75 | 3 | 2 | 66.7 | 1 | 0 | 0.0 | 1 | 0.2221 |
|  | ≧0.75 | 20 | 10 | 50.0 | 22 | 13 | 59.1 | 0.7569 |  |
| *DHFR* | ＜0.356 | 3 | 2 | 66.7 | 1 | 0 | 0.0 | 1 | 0.2221 |
|  | ≧0.356 | 20 | 10 | 50.0 | 22 | 13 | 59.1 | 0.7569 |  |
| *E2F1* | ＜0.373 | 14 | 9 | 64.3 | 13 | 7 | 53.8 | 0.7036 | 0.23 |
|  | ≧0.373 | 9 | 3 | 33.3 | 10 | 6 | 60.0 | 0.3698 |  |
| *BCL2L11* | ＜0.038 | 12 | 7 | 58.3 | 13 | 6 | 46.2 | 0.6951 | 0.2321 |
|  | ≧0.038 | 11 | 5 | 45.5 | 10 | 7 | 70.0 | 0.387 |  |
| *VEGFR* | ＜54.478 | 21 | 11 | 52.4 | 19 | 9 | 47.4 | 1 | 0.2389 |
|  | ≧54.478 | 2 | 1 | 50.0 | 4 | 4 | 100 | 0.3333 |  |
| *CCND1* | ＜55.178 | 19 | 11 | 57.9 | 15 | 8 | 53.3 | 1 | 0.2488 |
|  | ≧55.178 | 4 | 1 | 25.0 | 8 | 5 | 62.5 | 0.5454 |  |
| *SEMA5A* | ＜22.306 | 17 | 9 | 52.9 | 17 | 8 | 47.1 | 1 | 0.2561 |
|  | ≧22.306 | 6 | 3 | 50.0 | 6 | 5 | 83.3 | 0.5455 |  |
| *BAX* | ＜0.911 | 17 | 11 | 64.7 | 19 | 11 | 57.9 | 0.7419 | 0.2721 |
|  | ≧0.911 | 6 | 1 | 16.7 | 4 | 2 | 50.0 | 0.5 |  |
| *VCAM1* | ＜1.427 | 10 | 5 | 50.0 | 17 | 11 | 64.7 | 0.6868 | 0.283 |
|  | ≧1.427 | 13 | 7 | 53.8 | 6 | 2 | 33.3 | 0.6285 |  |
| *LGALS4* | ＜2.88 | 13 | 8 | 61.5 | 17 | 9 | 52.9 | 0.7213 | 0.2846 |
|  | ≧2.88 | 10 | 4 | 40.0 | 6 | 4 | 66.7 | 0.6084 |  |
| *MGMT* | ＜0.38 | 17 | 7 | 41.2 | 18 | 10 | 55.6 | 0.5051 | 0.2852 |
|  | ≧0.38 | 6 | 5 | 83.3 | 5 | 3 | 60.0 | 0.5455 |  |
| *RRM2* | ＜1.3 | 1 | 0 | 0.0 | 3 | 2 | 66.7 | 1 | 0.2868 |
|  | ≧1.3 | 22 | 12 | 54.5 | 20 | 11 | 55.0 | 1 |  |
| *OPRT* | ＜1.767 | 9 | 6 | 66.7 | 8 | 4 | 50.0 | 0.6372 | 0.2869 |
|  | ≧1.767 | 14 | 6 | 42.9 | 15 | 9 | 60.0 | 0.4661 |  |
| *SEC11A* | ＜47.614 | 21 | 11 | 52.4 | 20 | 10 | 50.0 | 1 | 0.2891 |
|  | ≧47.614 | 2 | 1 | 50.0 | 3 | 3 | 100 | 0.4 |  |
| *CLDN4* | ＜4.918 | 4 | 2 | 50.0 | 2 | 2 | 100 | 0.4667 | 0.2925 |
|  | ≧4.918 | 19 | 10 | 52.6 | 21 | 11 | 52.4 | 1 |  |
| *CDKN2A* | ＜3.919 | 21 | 12 | 57.1 | 19 | 11 | 57.9 | 1 | 0.2977 |
|  | ≧3.919 | 2 | 0 | 0.0 | 4 | 2 | 50.0 | 0.4667 |  |
| *PDCD5* | ＜0.488 | 1 | 1 | 100 | 6 | 3 | 50.0 | 1 | 0.3164 |
|  | ≧0.488 | 22 | 11 | 50.0 | 17 | 10 | 58.89 | 0.7479 |  |
| *ITGB3* | ＜0.13 | 18 | 10 | 55.6 | 19 | 10 | 52.69 | 1 | 0.3298 |
|  | ≧0.13 | 5 | 2 | 40.0 | 4 | 3 | 75.0 | 0.5238 |  |
| *SEMA3B* | ＜3.081 | 17 | 9 | 52.9 | 22 | 13 | 59.1 | 0.7535 | 0.3371 |
|  | ≧3.081 | 6 | 3 | 50.0 | 1 | 0 | 0.0 | 1 |  |
| *ESM1* | ＜0.079 | 20 | 10 | 50.0 | 18 | 11 | 61.1 | 0.5318 | 0.3641 |
|  | ≧0.079 | 3 | 2 | 66.7 | 5 | 2 | 40.0 | 1 |  |
| *FPGS* | ＜1.991 | 20 | 10 | 50.0 | 18 | 11 | 61.1 | 0.5318 | 0.3641 |
|  | ≧1.991 | 3 | 2 | 66.7 | 5 | 2 | 40.0 | 1 |  |
| *CADM1* | ＜0.05 | 3 | 3 | 100 | 4 | 3 | 75.0 | 1 | 0.4338 |
|  | ≧0.05 | 20 | 9 | 45.0 | 19 | 10 | 52.6 | 0.7524 |  |
| *KIAA1199* | ＜0.748 | 15 | 9 | 60.0 | 18 | 12 | 66.7 | 0.7307 | 0.4691 |
|  | ≧0.748 | 8 | 3 | 37.5 | 5 | 1 | 20.0 | 1 |  |
| *TK1* | ＜2.055 | 21 | 12 | 57.1 | 20 | 12 | 60.0 | 1 | 0.5331 |
|  | ≧2.055 | 2 | 0 | 0.0 | 3 | 1 | 33.3 | 1 |  |
| *ANGPT2* | ＜0.171 | 16 | 8 | 50.0 | 19 | 10 | 52.6 | 1 | 0.6812 |
|  | ≧0.171 | 7 | 4 | 57.1 | 4 | 3 | 75.0 | 1 |  |
| *GZMA* | ＜0.42 | 20 | 10 | 50.0 | 20 | 11 | 55.0 | 1 | 0.9128 |
|  | ≧0.42 | 3 | 2 | 66.7 | 3 | 2 | 66.7 | 1 |  |
