# Supplementary material for: Biomarker analysis to predict the pathological response to neoadjuvant chemotherapy in locally advanced gastric cancer: An exploratory biomarker study of COMPASS, a randomized phase II trial
Source: Oncotarget. 2020 Jul 28;11(30):2906–18. doi: 10.18632/oncotarget.27658 (PMC7392622; doi:10.18632/oncotarget.27658)
Supplement: Supplementary file 3 [file oncotarget-11-2906-s003.docx]

| **Supplementary Table 3: Relation between mRNA expression and clinicopathological features in pStage II/III gastric cancer**  **(different cohort, n=253).** | | | | | | | | | | | | | |
| --- | --- | --- | --- | --- | --- | --- | --- | --- | --- | --- | --- | --- | --- |
| **Variables/categories** | | ***ZDHHC14*** | | ***P*-value** |  | ***TIMP1*** | | ***P*-value** |  | ***CLDN18.2*** | | ***P*-value** |  |
|  |  | **≧ 0.608 (n=81)** | **＜0.608 (n=172)** |  |  | **≧10.473 (n=68)** | **< 10.473**  **(n=185)** |  |  | **≧23.564**  **(n=77)** | **＜23.564 (n=176)** |  |  |
| Age (years) |  |  |  |  |  |  |  |  |  |  |  |  |  |
| ＜67 |  | 43 | 89 | 0.842 |  | 35 | 97 | 0.892 |  | 43 | 89 | 0.4395 |  |
| ≧67 |  | 38 | 83 |  |  | 33 | 88 |  |  | 34 | 87 |  |  |
| Gender |  |  |  |  |  |  |  |  |  |  |  |  |  |
| Male |  | 57 | 113 | 0.4602 |  | 44 | 126 | 0.6094 |  | 48 | 122 | 0.2765 |  |
| Female |  | 24 | 59 |  |  | 24 | 59 |  |  | 29 | 54 |  |  |
| Histologic type |  |  |  |  |  |  |  |  |  |  |  |  |  |
| Differentiated | | 34 | 79 | 0.555 |  | 26 | 87 | 0.2124 |  | 28 | 85 | 0.079 |  |
| Undifferentiated | | 47 | 93 |  |  | 42 | 98 |  |  | 49 | 91 |  |  |
| Maximum tumor diameter | | | | |  |  |  |  |  |  |  |  |  |
| <65 mm |  | 41 | 94 | 0.5485 |  | 38 | 97 | 0.6258 |  | 40 | 95 | 0.7659 |  |
| ≧65 mm |  | 40 | 78 |  |  | 30 | 88 |  |  | 37 | 81 |  |  |
| Tumor depth | | | | |  |  |  |  |  |  |  |  |  |
| pT1 |  | 2 | 6 | 0.6755 |  | 2 | 6 | 0.6682 |  | 2 | 6 | 0.3694 |  |
| pT2 |  | 12 | 21 |  |  | 7 | 26 |  |  | 6 | 27 |  |  |
| pT3 |  | 28 | 50 |  |  | 20 | 68 |  |  | 27 | 51 |  |  |
| pT4 |  | 39 | 95 |  |  | 39 | 95 |  |  | 42 | 92 |  |  |
| Lymph node metastatic invasion | | | | |  |  |  |  |  |  |  |  |  |
| pN0 |  | 17 | 40 | 0.0699 |  | 20 | 37 | 0.0144 |  | 20 | 37 | 0.0192 |  |
| pN1 |  | 32 | 80 |  |  | 36 | 76 |  |  | 23 | 89 |  |  |
| pN2 |  | 18 | 17 |  |  | 6 | 29 |  |  | 15 | 20 |  |  |
| pN3 |  | 14 | 35 |  |  | 6 | 43 |  |  | 19 | 30 |  |  |
| pStage |  |  |  |  |  |  |  |  |  |  |  |  |  |
| II |  | 30 | 73 | 0.4143 |  | 29 | 74 | 0.704 |  | 31 | 72 | 0.9229 |  |
| III |  | 51 | 99 |  |  | 39 | 111 |  |  | 46 | 104 |  |  |
| Lymphatic invasion | | | | |  |  |  |  |  |  |  |  |  |
| Ly0 |  | 30 | 51 | 0.2401 |  | 20 | 61 | 0.5904 |  | 26 | 55 | 0.693 |  |
| Ly1 |  | 51 | 121 |  |  | 48 | 124 |  |  | 51 | 121 |  |  |
| Venous invasion | | | | |  |  |  |  |  |  |  |  |  |
| V0 |  | 27 | 46 | 0.2805 |  | 18 | 55 | 0.612 |  | 32 | 41 | **0.0032** |  |
| V1 |  | 54 | 126 |  |  | 50 | 130 |  |  | 45 | 135 |  |  |

| **Variables/categories** | | ***EGFR*** | | ***P*-value** |  | | ***RRM1*** | | | | | | ***P*-value** | | |  | ***MUC2*** | | | | | | | ***P*-value** | |
| --- | --- | --- | --- | --- | --- | --- | --- | --- | --- | --- | --- | --- | --- | --- | --- | --- | --- | --- | --- | --- | --- | --- | --- | --- | --- |
|  |  | **≧0.549**  **(n=62)** | **<0.549**  **(n=191)** |  |  | | **≧0.803**  **(n=141)** | | | **＜0.803 (n=112)** | | |  |  |  |  | **≧14.04 (n=104)** | | | | **＜14.04 (n=149)** | | |  |  |
| Age (years) |  |  |  |  |  | |  | | |  | | |  | | |  |  | | | |  | | |  | |
| ＜67 |  | 33 | 99 | 0.8487 |  | | 71 | | | 61 | | | 0.5157 | | |  | 52 | | | | 80 | | | 0.563 | |
| ≧67 |  | 29 | 92 |  |  | | 70 | | | 51 | | |  | | |  | 52 | | | | 69 | | |  | |
| Gender |  |  |  |  |  | |  | | |  | | |  | | |  |  | | | |  | | |  | |
| Male |  | 44 | 126 | 0.4663 |  | | 95 | | | 75 | | | 0.9448 | | |  | 75 | | | | 95 | | | 0.1636 | |
| Female |  | 18 | 65 |  |  | | 46 | | | 37 | | |  | | |  | 29 | | | | 54 | | |  | |
| Histologic type |  |  |  |  |  | |  | | |  | | |  | | |  |  | | | |  | | |  | |
| Differentiated | | 30 | 83 | 0.4973 |  | | 70 | | | 43 | | | 0.0737 | | |  | 56 | | | | 57 | | | 0.0141 | |
| Undifferentiated | | 32 | 108 |  |  | | 71 | | | 69 | | |  | | |  | 48 | | | | 92 | | |  | |
| Maximum tumor diameter | | | | |  | |  | | |  | | |  | | |  |  | | | |  | | |  | |
| <65 mm |  | 34 | 101 | 0.7882 |  | | 69 | | | 66 | | | 0.1135 | | |  | 58 | | | | 77 | | | 0.521 | |
| ≧65 mm |  | 28 | 90 |  |  | | 72 | | | 46 | | |  | | |  | 46 | | | | 72 | | |  | |
| Tumor depth | | | | |  | |  | | |  | | |  | | |  |  | | | |  | | |  | |
| pT1 |  | 2 | 6 | 0.6338 |  | | 6 | | | 2 | | | 0.7112 | | |  | 2 | | | | 6 | | | 0.2582 | |
| pT2 |  | 9 | 24 |  |  | | 19 | | | 14 | | |  | | |  | 16 | | | | 17 | | |  | |
| pT3 |  | 15 | 63 |  |  | | 42 | | | 36 | | |  | | |  | 37 | | | | 41 | | |  | |
| pT4 |  | 36 | 98 |  |  | | 74 | | | 60 | | |  | | |  | 49 | | | | 85 | | |  | |
| Lymph node metastasis | | | | |  | |  | | |  | | |  | | |  |  | | | |  | | |  | |
| pN0 |  | 11 | 46 | **<0.001** |  | | 33 | | | 24 | | | 0.0197 | | |  | 25 | | | | 32 | | | 0.4279 | |
| pN1 |  | 43 | 69 |  |  | | 72 | | | 40 | | |  | | |  | 49 | | | | 63 | | |  | |
| pN2 |  | 2 | 33 |  |  | | 17 | | | 18 | | |  | | |  | 10 | | | | 25 | | |  | |
| pN3 |  | 6 | 43 |  |  | | 19 | | | 30 | | |  | | |  | 20 | | | | 28 | | |  | |
| pStage |  |  |  |  |  | | |  | | |  | | |  | | | | |  | | |  | | |  |
| II |  | 22 | 81 | 0.3349 |  | 60 | | | 43 | | | 0.5035 | | |  | | | 44 | | 59 | | | 0.6659 | |  |
| III |  | 40 | 110 |  |  | 81 | | | 69 | | |  | | |  | | | 60 | | 90 | | |  | |  |
| Lymphatic invasion | |  |  |  |  |  | | |  | | |  | | |  | | |  | |  | | |  | |  |
| Ly0 |  | 22 | 59 | 0.5005 |  | 48 | | | 33 | | | 0.4382 | | |  | | | 33 | | 48 | | | 0.9353 | |  |
| Ly1 |  | 40 | 132 |  |  | 93 | | | 79 | | |  | | |  | | | 71 | | 101 | | |  | |  |
| Venous invasion | |  |  |  |  |  | | |  | | |  | | |  | | |  | |  | | |  | |  |
| V0 |  | 11 | 62 | **0.0262** |  | 42 | | | 31 | | | 0.5561 | | |  | | | 20 | | 53 | | | **0.0048** | |  |
| V1 |  | 51 | 129 |  |  | 99 | | | 81 | | |  | | |  | | | 84 | | 96 | | |  | |  |

| **Variables/categories** | | | | ***DSG2*** | | | | ***P*-value** | | |
| --- | --- | --- | --- | --- | --- | --- | --- | --- | --- | --- |
|  | |  | | **≧4.312 (n=151)** | | **<4.312 (n=102)** | |  |  |  |
| Age (years) |  | |  | |  | |  | |  |  |
| ＜67 |  | | 72 | | 60 | | 0.0813 | |  |  |
| ≧67 |  | | 79 | | 42 | |  | |  |  |
| Gender |  | |  | |  | |  | |  |  |
| Male |  | | 100 | | 70 | | 0.6897 | |  |  |
| Female |  | | 51 | | 32 | |  | |  |  |
| Histologic type |  | |  | |  | |  | |  |  |
| Differentiated | | | 81 | | 32 | | **0.0005** | |  |  |
| Undifferentiated | | | 70 | | 70 | |  | |  |  |
| Maximum tumor diameter | | | | | | | | | |  |
| <65 mm |  | | 76 | | 59 | | 0.24 | |  |  |
| ≧65 mm |  | | 75 | | 43 | |  | |  |  |
| Tumor depth | | | | | | | | | |  |
| pT1 |  | | 5 | | 3 | | **0.0414** | |  |  |
| pT2 |  | | 22 | | 11 | |  | |  |  |
| pT3 |  | | 55 | | 23 | |  | |  |  |
| pT4 |  | | 69 | | 65 | |  | |  |  |
| Lymph node metastasis | | | | | | | | | |  |
| pN0 |  | | 35 | | 22 | | 0.8243 | |  |  |
| pN1 |  | | 64 | | 48 | |  | |  |  |
| pN2 |  | | 23 | | 12 | |  | |  |  |
| pN3 |  | | 29 | | 20 | |  | |  |  |
| pStage |  | |  | |  | |  | |  |  |
| II |  | | 64 | | 38 | | 0.5076 | |  |  |
| III |  | | 87 | | 64 | |  | |  |  |
| Lymphatic invasion | | |  |  |  |  |  |  |  |  |
| Ly0 |  | | 50 | | 31 | | 0.6491 | |  |  |
| Ly1 |  | | 101 | | 71 | |  | |  |  |
| Venous invasion | | |  |  |  |  |  |  |  |  |
| V0 |  | | 43 | | 30 | | 0.8721 | |  |  |
| V1 |  | | 108 | | 72 | |  | |  |  |

pStage: pathological Stage
